# Updated Information on the Epidemiology of Middle East Respiratory Syndrome Coronavirus (MERS-CoV) Infection and Guidance for the Public, Clinicians, and Public Health Authorities, 2012–2013

**Published:** 2013-09-27

**Authors:** Div of Global Migration and Quarantine, Div of Healthcare Quality Promotion, National Center for Emerging and Zoonotic Infectious Diseases, Div of Global Health Protection (proposed), Center for Global Health, Div of Viral Diseases, National Center for Immunization and Respiratory Diseases, CDC

The Middle East respiratory syndrome coronavirus (MERS-CoV) was first reported to cause human infection in September 2012 (1). In July 2013, the World Health Organization (WHO) International Health Regulations Emergency Committee determined that MERS-CoV did not meet criteria for a “public health emergency of international concern,” but was nevertheless of “serious and great concern” (2). This report summarizes epidemiologic information and provides updates to CDC guidance about patient evaluation, case definitions, travel, and infection control as of September 20, 2013.

As of September 20, 2013, a total of 130 cases from eight countries have been reported to WHO; 58 (45%) of these cases have been fatal (Figure 1). All cases have been directly or indirectly linked through travel to or residence in four countries: Saudi Arabia, Qatar, Jordan, and the United Arab Emirates (UAE) (Figure 2). The median age of persons with confirmed MERS-CoV infection is 50 years (range: 2–94 years). The male-to-female ratio is 1.6 to 1.0. Twenty-three (18%) of the cases occurred in persons who were identified as health-care workers. Although most reported cases involved severe respiratory illness requiring hospitalization, at least 27 (21%) involved mild or no symptoms. Despite evidence of person-to-person transmission, the number of contacts infected by persons with confirmed infections appears to be limited. No cases have been reported in the United States, although 82 persons from 29 states have been tested for MERS-CoV infection.

Potential animal reservoirs and mechanism(s) of transmission of MERS-CoV to humans remain unclear. A zoonotic origin for MERS-CoV was initially suggested by high genetic similarity to bat coronaviruses (3), and some recent reports have described serologic data from camels and the identification of related viruses in bats (4–6). However, more epidemiologic data linking cases to infected animals are needed to determine if a particular species is a host, a source of human infection, or both.

To date, the largest, most complete clinical case series published included 47 patients; most had fever (98%), cough (83%), and shortness of breath (72%). Many also had gastrointestinal symptoms (26% had diarrhea, and 21% had vomiting). All but two patients (96%) had one or more chronic medical conditions, including diabetes (68%), hypertension (34%), heart disease (28%), and kidney disease (49%). Thirty-four (72%) had more than one chronic condition (7). Nearly half the patients in this series were part of a health-care–associated outbreak in Al-Ahsa, Saudi Arabia (i.e., a population that would be expected to have high rates of underlying conditions) (8). Also, the prevalence of diabetes in persons aged ≥50 years in Saudi Arabia has been reported to be nearly 63% (9). It remains unclear whether persons with specific conditions are disproportionately infected with MERS-CoV or have more severe disease.

## CDC Guidance

### Evaluating patients

CDC has changed its guidance to indicate that testing for MERS-CoV and other respiratory pathogens* can be conducted simultaneously and that positive results for another respiratory pathogen should not necessarily preclude testing for MERS-CoV. Health-care providers in the United States should continue to evaluate patients for MERS-CoV infection if they develop fever and pneumonia or acute respiratory distress syndrome (ARDS) within 14 days after traveling from countries in or near the Arabian Peninsula.† Providers also should evaluate patients for MERS-CoV infection if they have ARDS or fever and pneumonia, and have had close contact§ with a recent traveler from this area who has fever and acute respiratory illness.

CDC continues to recommend that clusters¶ of patients with severe acute respiratory illness (e.g., fever and pneumonia requiring hospitalization) be evaluated for common respiratory pathogens and reported to local and state public health departments. If the illnesses remain unexplained, particularly if the cluster includes health-care providers, testing for MERS-CoV should be considered, in consultation with state and local health departments. In this situation, testing should be considered even for patients without travel-related exposure. Additional information about CDC’s interim guidance regarding who should be evaluated for MERS-CoV infection is available at http://www.cdc.gov/coronavirus/mers/interim-guidance.html.

### Case definitions

Although CDC has not changed the case definition of a confirmed case, confirmatory laboratory testing now requires a positive polymerase chain reaction of at least two, instead of one, specific genomic targets or a single positive target with sequencing of a second. CDC’s definition of a probable case has been changed so that identification of another etiology does not exclude a person with an illness meeting this definition from being classified as having a probable case. Additional information about CDC’s case definitions is available at http://www.cdc.gov/coronavirus/mers/case-def.html.

### Travel guidance

The peak travel season to Saudi Arabia is July through November, coinciding with the religious pilgrimages of Hajj and Umrah. CDC encourages pilgrims to consider recommendations from the Saudi Arabia Ministry of Health regarding persons who should postpone their pilgrimages this year, including persons aged ≥65 years, children, pregnant women, and persons with chronic diseases, weakened immune systems, or cancer (http://www.moh.gov.sa/en/coronanew/news/pages/news-2013-7-14-001.aspx). WHO advises that persons with preexisting medical conditions consult a health-care provider before deciding whether to make a pilgrimage (http://www.who.int/ith/updates/20130725/en).

CDC continues to recommend that U.S. travelers to countries in or near the Arabian Peninsula protect themselves from respiratory diseases, including MERS-CoV, by washing their hands often and avoiding contact with persons who are ill. If travelers to the region have onset of fever with cough or shortness of breath during their trip or within 14 days of returning to the United States, they should seek medical care. They should tell their health-care provider about their recent travel. More detailed travel recommendations related to MERS-CoV are available at http://wwwnc.cdc.gov/travel/notices/watch/coronavirus-arabian-peninsula.

### Infection control

With multiple health-care–associated clusters identified (8,10), infection control remains a primary means of preventing and controlling MERS-CoV transmission. CDC has recently made checklists available that highlight key actions that health-care providers and facilities can take to prepare for MERS-CoV patients (http://www.cdc.gov/coronavirus/mers/preparedness/index.html). CDC’s infection control guidance has not changed. Standard, contact, and airborne precautions are recommended for management of hospitalized patients with known or suspected MERS-CoV infection.

CDC has determined that federal isolation and quarantine are authorized for MERS-CoV under Executive Order 13295 (http://www.cdc.gov/quarantine/aboutlawsregulationsquarantineisolation.html).** At this time, CDC is not restricting the movement of travelers with respiratory illness (that is not confirmed or probable MERS-CoV infection) arriving from the Arabian Peninsula. However, persons with illness meeting CDC’s definition of a confirmed or probable case of MERS-CoV infection should remain in isolation until they are no longer considered to be contagious according to current guidance. Those who do not adhere to isolation requirements, or who intend to travel, may be subject to additional public health measures. CDC does not recommend quarantine of asymptomatic persons who were exposed to confirmed or probable cases. CDC generally recommends that persons with febrile respiratory illness delay travel until their symptoms resolve.

CDC has issued new guidance for care and management of MERS-CoV patients in the home and guidance for close contacts of these patients (http://www.cdc.gov/coronavirus/mers/hcp/home-care.html). Persons who are confirmed, or being evaluated for MERS-CoV infection, and do not require hospitalization for medical reasons should be isolated in their homes as long as the home is deemed suitable for isolation. CDC currently recommends MERS-CoV patients should be isolated at home until public health authorities or a health-care provider determine that they are no longer contagious. Persons who might have been exposed†† to MERS-CoV should be monitored for fever and respiratory symptoms for 14 days after the most recent exposure. Asymptomatic exposed persons do not need to limit their activities outside the home. If persons exposed to MERS-CoV have onset of symptoms, they should contact a health-care provider as soon as possible and follow the precautions for limiting possible exposure of other persons to MERS-CoV.

More detailed MERS-CoV–related interim guidance about patient evaluation, case definitions, travel, and infection control is available at http://www.cdc.gov/coronavirus/mers/index.html. This guidance might change as CDC learns more about the epidemiology of MERS-CoV. CDC will continue to post the most current information and guidance on its MERS-CoV website. State and local health departments with questions should contact the CDC Emergency Operations Center at 770-488-7100.

What is already known on this topic?

The Middle East respiratory syndrome coronavirus (MERS-CoV) was first reported to cause human infection in September 2012 and is associated with high death rates. All cases have been linked through travel to or residence in Saudi Arabia, Qatar, Jordan, and United Arab Emirates. No cases have been reported in the United States.

What is added by this report?

This report summarizes epidemiologic information about MERS-CoV, provides updates to CDC guidance about patient evaluation, case definitions, travel, and infection control as of September 20, 2013, and describes new guidance for home care and management of patients with MERS-CoV infection.

What are the implications for public health practice?

Cases of MERS-CoV infection continue to be reported by countries in and near the Arabian Peninsula. This updated CDC guidance will help health-care providers and state and local health departments prepare for and respond to a possible case in the United States.

## Figures and Tables

**FIGURE 1:**
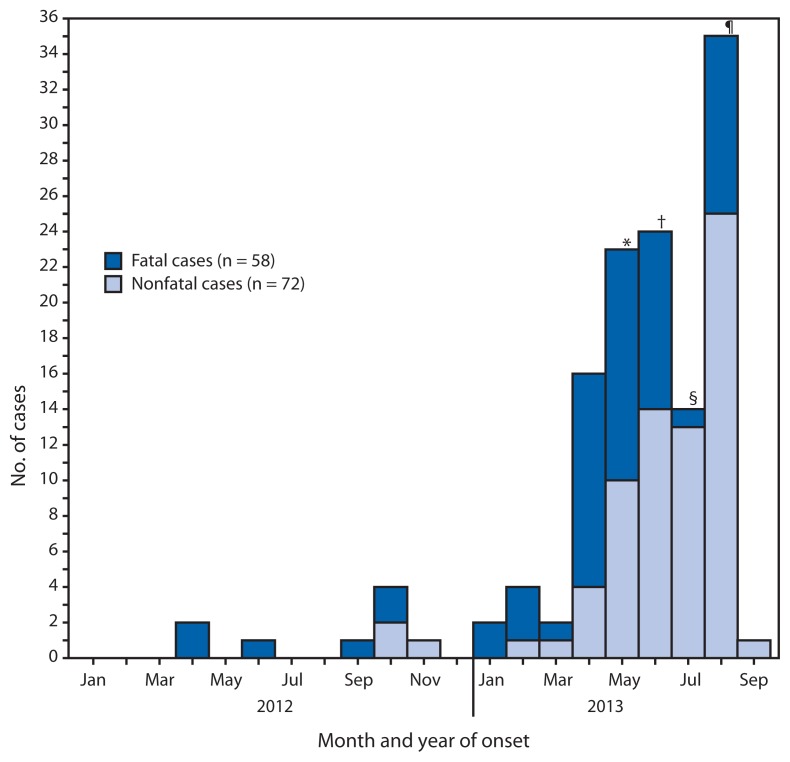
Number of cases of Middle East respiratory syndrome coronavirus infection (58 fatal and 72 nonfatal) reported to the World Health Organization (WHO) as of September 20, 2013, by month of illness onset — worldwide, 2012–2013 * Case count for May assumes that three cases included in WHO announcements on May 22, May 23, and June 2, 2013, had symptom onset during May 2013. ^†^ Case count for June assumes that 22 cases included in WHO announcements on June 14, June 17, June 22, June 23, June 26, July 5, July 7, and July 11, 2013, had symptom onset during June 2013. ^§^ Case count for July assumes that 10 cases included in WHO announcements on July 18, July 21, and August 1, 2013, had symptom onset during July 2013. ^¶^ Case count for August assumes that 25 cases (two on August 28, one on August 29, two on August 30, and 16 on September 16) had symptom onset during August 2013.

**FIGURE 2:**
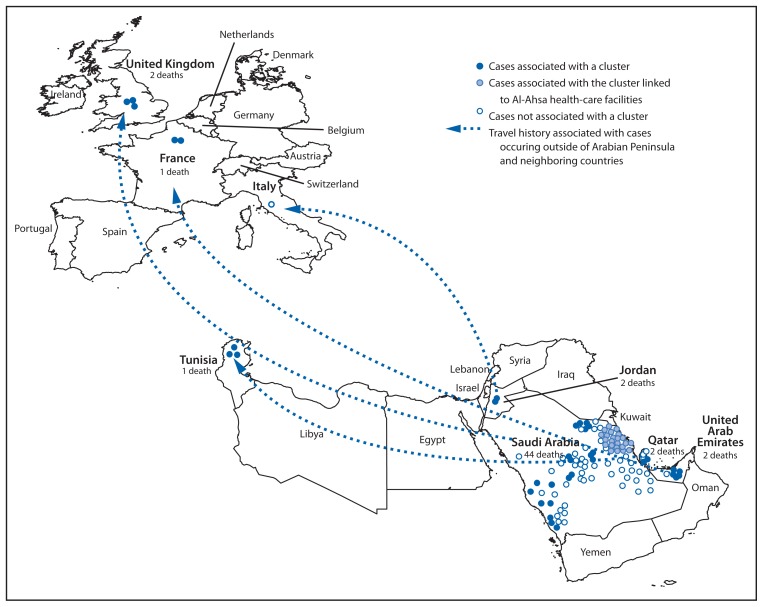
Confirmed cases of Middle East respiratory syndrome coronavirus infection (N = 130) reported to the World Health Organization as of September 20, 2013, and history of travel from in or near the Arabian Peninsula* within 14 days of illness onset — worldwide, 2012–2013 * Dots are not geographically representative of exact location of residences of persons with infection.

## Footnotes

* Examples of respiratory pathogens causing community-acquired pneumonia include influenza A and B, respiratory syncytial virus, *Streptococcus pneumoniae*, and *Legionella pneumophila*.

† Countries considered in or near the Arabian Peninsula include Bahrain, Iraq, Iran, Israel, Jordan, Kuwait, Lebanon, Oman, Palestinian Territories, Qatar, Saudi Arabia, Syria, UAE, and Yemen.

§ Close contact is defined as 1) any person who provided care for the patient, including a health-care worker or family member, or had similarly close physical contact; or 2) any person who stayed at the same place (e.g., lived with or visited) as the patient while the patient was ill.

¶ In accordance with WHO guidance for MERS-CoV, a cluster is defined as “two or more persons with onset of symptoms within the same 14-day period who are associated with a specific setting, such as a classroom, workplace, household, extended family, hospital, other residential institution, military barracks, or recreational camp.” Information available at http://www.who.int/csr/disease/coronavirus_infections/InterimRevisedSurveillanceRecommendations_nCoVinfection_27Jun13.pdf.

** Severe acute respiratory syndrome (SARS) was added to Executive Order 13295 (http://www.gpo.gov/fdsys/pkg/WCPD-2003-04-07/pdf/WCPD-2003-04-07-Pg408.pdf) in response to the 2003 outbreak. SARS is defined under the executive order as a disease associated with fevers and signs and symptoms of pneumonia or other respiratory illness, transmitted from person-to-person predominantly by the aerosolized or droplet route, and, if spread in the population, would have severe public health consequences. MERS-CoV infection meets these syndromic criteria and therefore meets the criteria for a quarantinable communicable disease. SARS and MERS-CoV infections are caused by different but related coronaviruses.

†† Persons who might have been exposed to MERS-CoV include persons who care for or have close contact with someone who has MERS-CoV infection, persons who recently traveled to countries in or near the Arabian Peninsula, and persons who were identified as a result of a public health investigation of MERS-CoV infection cases.
